# MiR-324-3p promotes tumor growth through targeting DACT1 and activation of Wnt/β-catenin pathway in hepatocellular carcinoma

**DOI:** 10.18632/oncotarget.20058

**Published:** 2017-08-07

**Authors:** Hang Tuo, Yufeng Wang, Liang Wang, Bowen Yao, Qing Li, Cong Wang, Zhikui Liu, Shaoshan Han, Guozhi Yin, Kangsheng Tu, Qingguang Liu

**Affiliations:** ^1^ Department of Hepatobiliary Surgery, The First Affiliated Hospital of Xi’an Jiaotong University, Xi’an, Shaanxi Province 710061, China

**Keywords:** hepatocellular carcinoma, miR-324-3p, DACT1, proliferation, Wnt/β-catenin pathway

## Abstract

Recently, it has been reported that miR-324-3p participates in regulation of the carcinogenesis and tumor progression in various cancers. However, the expression and function of miR-324-3p in hepatocellular carcinoma (HCC) remain unclear. In the current study, miR-324-3p expression was significantly up-regulated in HCC tissues and cell lines. HCC patients with high miR-324-3p level showed poor prognostic features and shorter overall survival and disease-free survival. And *in vitro* and *in vivo* experiments revealed that miR-324-3p promoted cell viability, colony formation, proliferation and cell cycle progression of HCC cells. Further studies demonstrated that miR-324-3p could directly target DACT1 (dishevelled binding antagonist of beta catenin 1) and negatively regulated its expression in HCC cells. And rescue experiments revealed that DACT1 could reverse the effects of miR-324-3p on HCC cells. Furthermore, the accumulation of both cytoplasmic and nuclear β-catenin as well as its downstream targets including c-Myc and cyclin D1 could be positively regulated by miR-324-3p. The regulatory effects of miR-324-3p on β-catenin, c-Myc and cyclin D1 levels could be reversed by DACT1. Overall, we concluded that miR-324-3p could promote tumor growth through targeting DACT1 and activation of Wnt/β-catenin pathway in HCC. MiR-324-3p may be a ponderable and promising therapeutic target for HCC.

## INTRODUCTION

Hepatocellular carcinoma (HCC), which results in significant high mortality of patients, is one of the most common cancers in the world [1, 2]. Despite great improvements have been achieved in diagnosis and therapy of HCC over the past few decades, the prognosis of HCC patients remains poor [1, 2]. Thus, it is urgent for us to explore the molecular mechanisms underlying HCC occurrence and development, which will contribute to the improvements of novel therapeutic strategies.

MicroRNAs (miRNAs) are small noncoding RNAs (18–25 nt), which have been identified to play a critical role in various biological processes of cancer cells by regulating target gene expression at the post-transcriptional level [3–6]. For example, it has been reported that miR-212 suppresses tumor growth of HCC by targeting forkhead box protein A1 (FOXA1) [7]. And miR-15b-5p regulates HCC growth and metastasis through the AKT/mTORC1 and β-catenin signaling pathways [8]. Recently, Liu C *et al.* reported that miR-324-3p could modulate cancer cell growth and apoptosis by targeting SMAD family member 7 (SMAD7) in nasopharyngeal carcinoma [9]. Gao X *et al.* demonstrated that up-regulated miR-324-3p expression was an independent prognostic predictor for early stage lung squamous cell carcinoma [10]. Notably, previous study reported that plasma miR-324-3p could be used as a preclinical biomarker for HCC. However, rare research investigates the expression, functions and mechanisms of miR-324-3p in HCC.

Wnt/β-catenin signaling pathway, which is the canonical Wnt pathway, plays a critical role in the proliferation and cell cycle progression of HCC cells [11–13]. The activation of this pathway initiates from Wnt proteins binding to the N-terminal extra-cellular cysteine-rich domain of a Frizzled (Fz) family and co-receptor LDL receptor related protein (LRP)-5/6, receptor tyrosine kinase (RTK) and receptor tyrosine kinase like orphan receptor 2 (ROR2). Ligand binding to the receptor leads to the phosphorylation of Dishevelled (Dvl), which recruits AXIN and glycogen synthase kinase-3β (GSK3β) to the cell membrane. AXIN/GSK3β complex subsequently inhibits the phosphorylation of β-catenin and leads to β-catenin dissociating from the destruction complex, which causes an accumulation of β-catenin in the cytoplasm and its eventual translocation into the nucleus. Next, the β-catenin accumulation in the nucleus interacts with the TCF/LEF to transcriptionally activate downstream gene expression, such as cyclin D, c-Myc [14]. Increasing evidences reveal that DACT1(dishevelled binding antagonist of beta catenin 1), also called HDPR1, functions as an inhibitor of Wnt signaling through its interaction with Dvl, a central mediator of Wnt pathways [15–18]. For example, recent study in HCC demonstrates that the decreased DACT1 expression leads to accumulation of both cytoplasmic and nuclear β-catenin, which results in activation of Wnt/β-catenin signaling and promotes HCC progression [18].

Here, we reported that miR-324-3p was up-regulated in HCC, and highly expressed miR-324-3p was significantly associated with the malignant clinicopathologic features and poor prognosis of HCC patients. Functionally, up-regulated miR-324-3p expression promoted cell viability, colony formation, proliferation and cell-cycle progression in HCC. Furthermore, DACT1, an inhibitor of Wnt/β-catenin signaling pathway, was determined as a direct target of miR-324-3p in HCC. And miR-324-3p could exert its oncogenic role possibly by activating Wnt/β-catenin signaling pathway. Taken together, elevated miR-324-3p expression promotes HCC growth by inhibiting the expression of DACT1 and subsequently activating Wnt/β-catenin signaling pathway.

## RESULTS

### MiR-324-3p is increased in HCC

**Figure 1:**
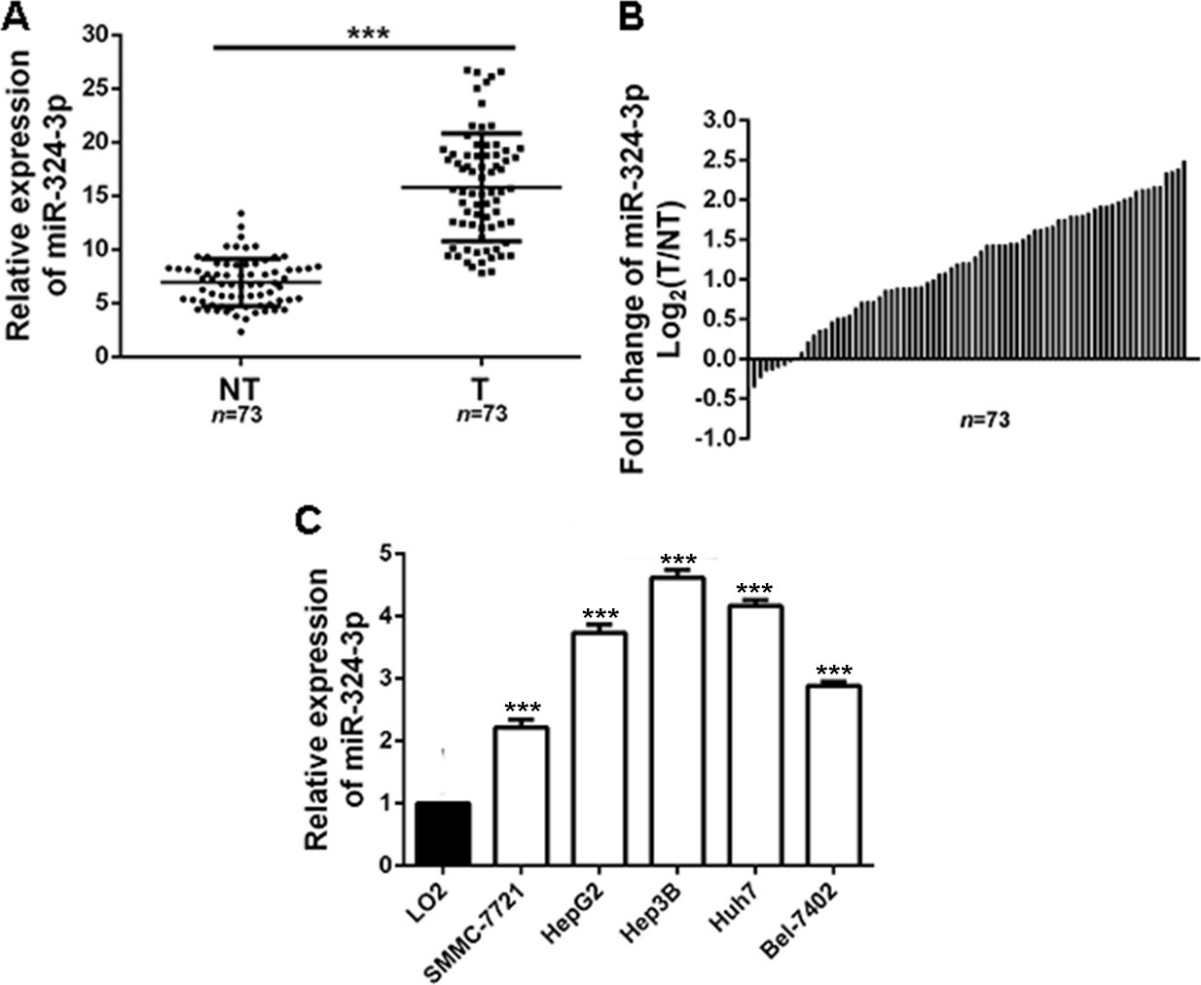
MiR-324-3p is frequently upregulated in HCC tissues and cell lines (**A**, **B**) Relative expression levels of miR-324-3p in 73 paired HCC tissues (T) and adjacent nontumor tissues (NT) were determined by real-time PCR. *n* = 73, ***P < 0.001 by *t* test. (**C**) The expression of miR-324-3p in five HCC cell lines were significantly increased compared to LO2 cells. *n* = three repeats with similar results, ***P < 0.001 by ANOVA.

Firstly, we measured the expression of miR-324-3p in HCC tissues and adjacent nontumor tissues. The results showed that the expression of miR-324-3p was increased significantly in HCC tissues compared to the control groups (*P* < 0.001, Figure 1A and 1B). Furthermore, the expression of miR-324-3p was detected in HCC cell lines. Consistently, we observed that miR-324-3p expression was notably up-regulated in HCC cell lines compared with LO2 (*P* < 0.05, Figure 1C). Hep3B cells had the highest expression while SMMC-7721 showed the lowest expression of miR-324-3p (Figure 1C). And these two cell lines were selected for the subsequent experiments. Taken together, these data suggested that miR-324-3p might be an oncogene in HCC.

### The clinical significance of elevated miR-324-3p expression in HCC patients

**Figure 2:**
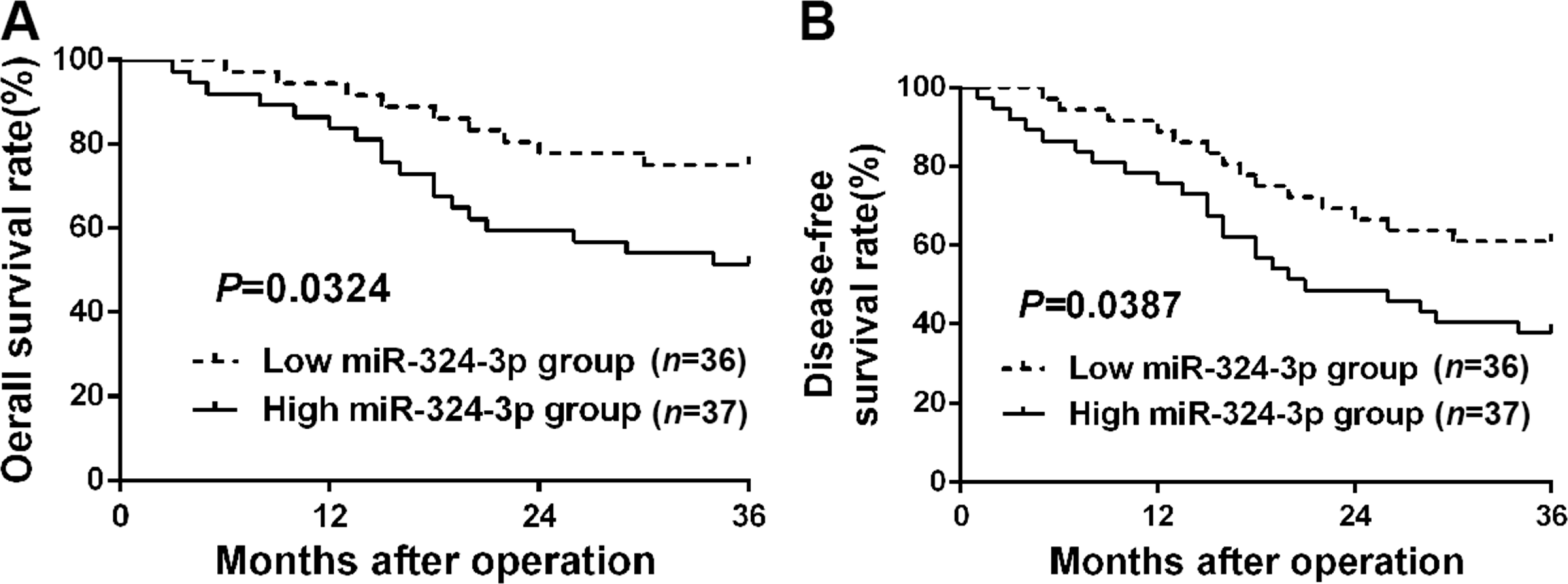
The prognostic significance of miR-324-3p in HCC patients All HCC patients were grouped into low miR-324-3p group (*n* = 36) and high miR-324-3p group (*n* = 37) according to the median level of miR-324-3p. HCC patients with high miR-324-3p level had significant shorter (**A**) overall survival (OS) and (**B**) disease-free survival (DFS). *P* < 0.05 by Log-rank test.

To explore the clinical significance of miR-324-3p in HCC, 73 patients were sorted into two groups according to cutoff value, which was defined as the median expression level of miR-324-3p. As showed in Table 1, high expression of miR-324-3p was closely correlated with large tumor size(*P* = 0.003), multiple tumor nodules (*P* = 0.007) and advanced TNM tumor stage (*P* = 0.020). In addition, Kaplan–Meier survival curves suggested that HCC patients in high miR-324-3p group had obvious shorter 3-year overall survival (OS) and disease-free survival (DFS) (*P* < 0.05, Figure 2A and 2B) compared to those in low miR-324-3p group. In conclusion, these data suggested miR-324-3p as a predictive biomarker for the prognosis of HCC patients.

**Table 1:** Correlation between expression of miR-324-3p and the clinicopathologic characteristics in HCC (*n* = 73)

Characteristics		Cases (*n =* 73)	Expression level of miR-324-3p		*P*
			high (*n =* 37)	low (*n =* 36)	
Age (year)	< 50	23	13	10	0.499
	≥ 50	50	24	26	
Gender	Male	62	32	30	0.707
	Female	11	5	6	
HBV	Absent	12	7	5	0.562
	Present	61	30	31	
Serum AFP level (ng/mL)	< 400	16	9	7	0.614
	≥ 400	57	28	29	
**Tumor size (cm)**	< 5	32	10	22	0.003***
	≥ 5	41	27	14	
**Number of** **tumor nodules**	1	60	26	34	0.007***
	≥ 2	13	11	2	
Cirrhosis	Absent	18	8	10	0.542
	Present	55	29	26	
Venous infiltration	Absent	52	23	29	0.083
	Present	21	14	7	
Edmondson-Steiner grading	I + II	47	21	26	0.168
	III + IV	26	16	10	
**TNM tumor stage**	I + II	54	23	31	0.020*
	III + IV	19	14	5	

HCC, hepatocellular carcinoma; HBV, hepatitis B virus;

AFP, alpha-fetoprotein; TNM, tumor-node-metastasis.

*P < 0.05, ***P < 0.001.

### MiR-324-3p promotes cell viability, colony formation, proliferation and cell cycle progression in HCC

**Figure 3:**
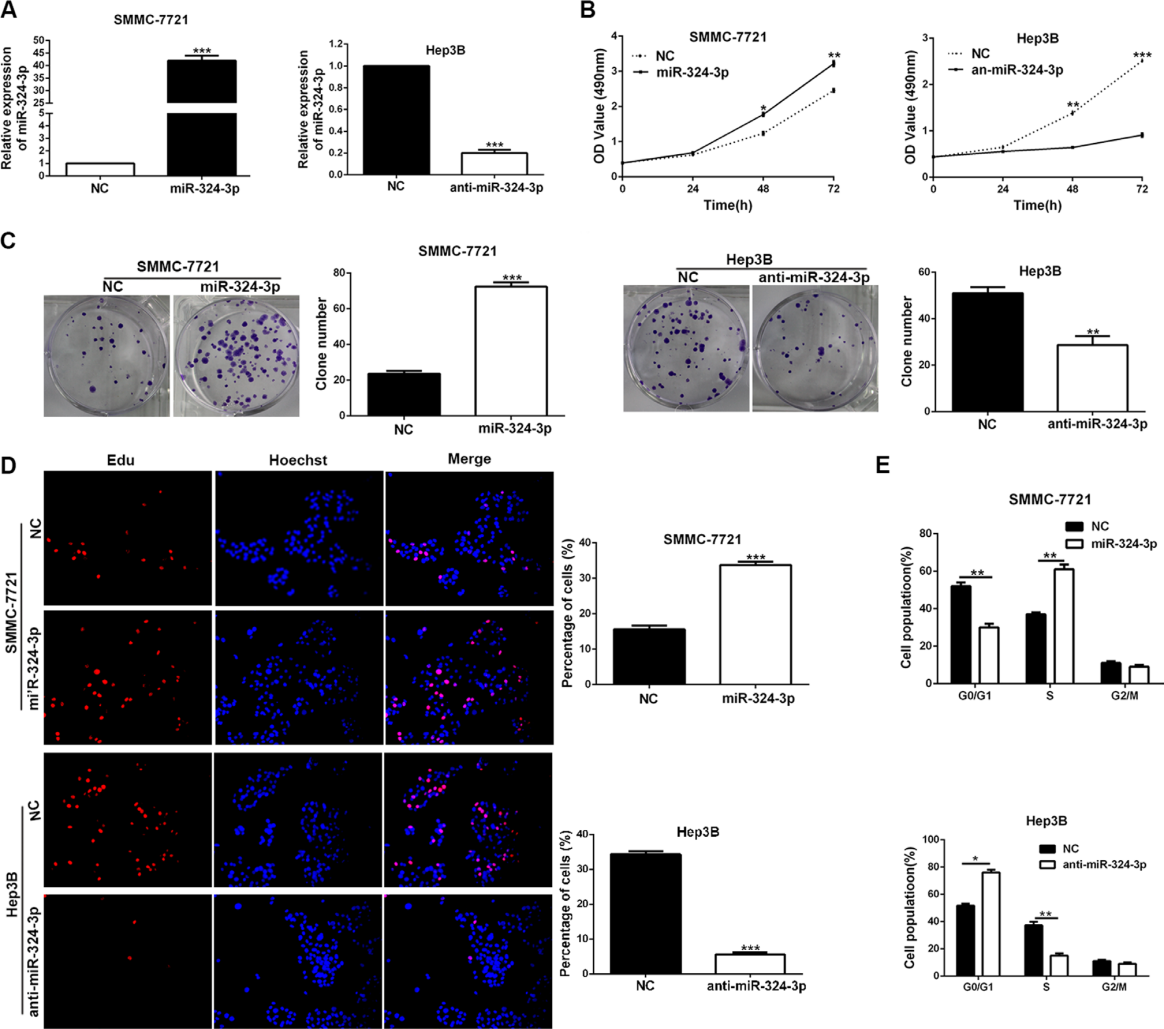
MiR-324-3p promotes cell viability, colony formation, proliferation and cell-cycle progression in HCC cells (**A**) The expression changes of miR-324-3p was measured in SMMC-7721 and Hep3B cells after being transfected with miR-324-3p mimics or inhibitors. *n* = three repeats with similar results, ***P < 0.001 by t test. (**B**) Cell viability was evaluated by the MTT assay at the indicated days. miR-324-3p overexpression promoted while its silencing inhibited viability of HCC cells. *n* = three repeats with similar results, *P < 0.05, **P < 0.01 and ***P < 0.001 by ANOVA. (**C**) Representative results for colony formation by the indicated cells. The number of cell colonies were increased after miR-324-3p overexpression while reduced after miR-324-3p knockdown. *n* = three repeats with similar results, **P < 0.01 and ***P < 0.001 by *t* test. (**D**) Representative micrographs (left) and quantification (right) of BrdU incorporation assay of SMMC-7721 and Hep3B cells. miR-324-3p positively regulated proliferation of HCC cells. Original magnification was 400×. *n* = three repeats with similar results, ***P < 0.001 by *t* test. (**E**) Modulating miR-324-3p levels regulated the cell cycle progression of HCC cells as measured by flow cytometry analysis. *n* = three repeats with similar results, *P < 0.05 and **P < 0.01 by *t* test.

In order to determine the functions of miR-324-3p in HCC cells, we transfected miR-324-3p mimics into SMMC-7721 cells, while miR-324-3p inhibitors were transfected into Hep3B cells. Then the expression changes of miR-324-3p were detected by real-time PCR (*P* < 0.001, Figure 3A). Results of MTT assay, colony formation assay, EdU cell proliferation assay and cell cycle analysis revealed that up-regulation of miR-324-3p markedly promoted cell viability, colony formation, proliferation and cell cycle progression in SMMC-7721 cells (*P* < 0.05, Figure 3B–3F). In accordance, miR-324-3p knockdown inhibited these cellular processes of Hep3B cells (*P* < 0.05, Figure 3B–3F).

**Figure 4:**
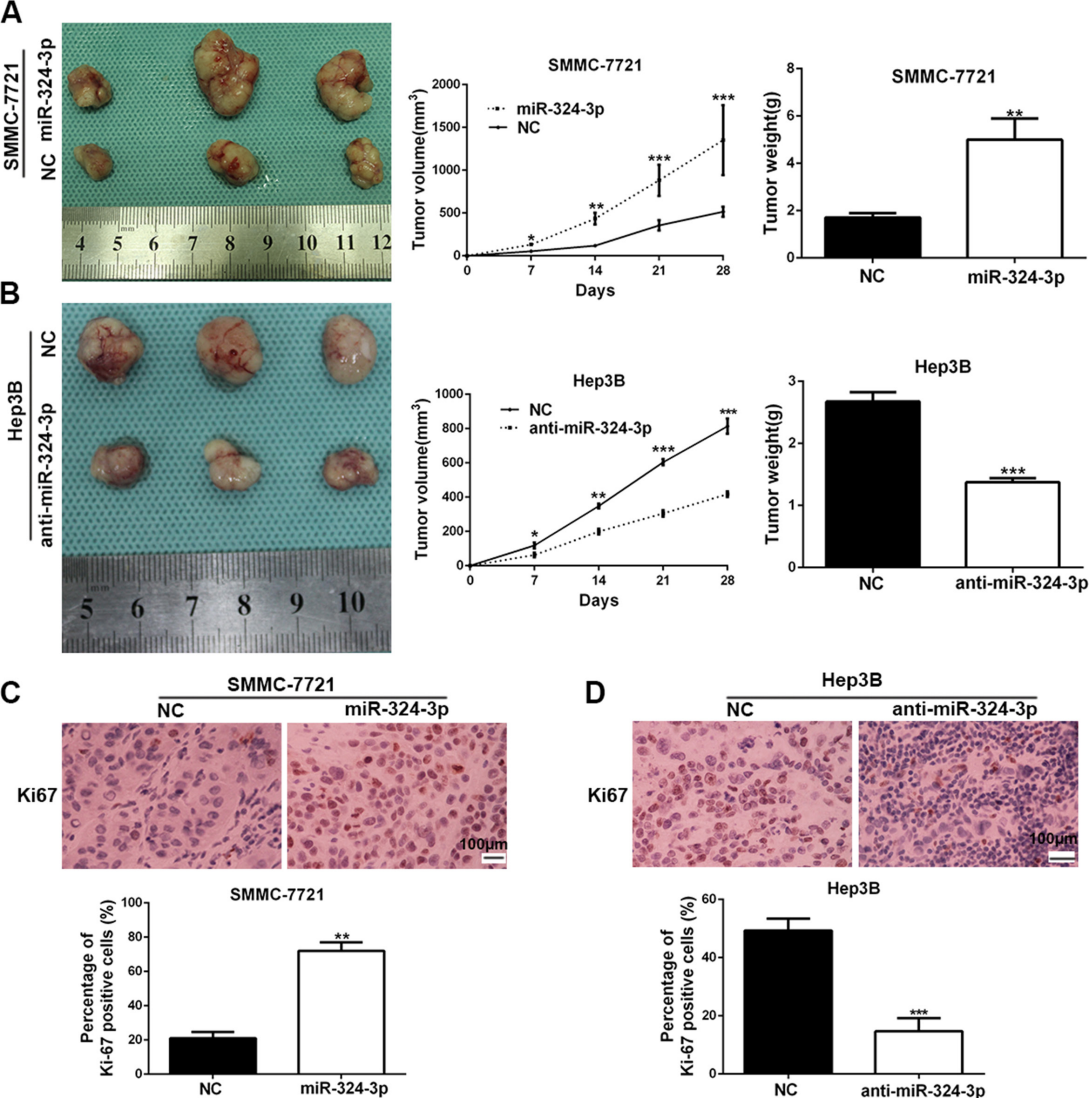
MiR-324-3p promotes HCC growth *in vivo* (**A**) Nude mice were respectively subcutaneously transplanted with Lv-NC-SMMC-7721 cells (*n* = 3) or Lv-miR-324-3p-SMMC-7721 cells (*n* = 3, left panel). Tumor volume (middle panel) and weight (right panel) revealed that increased expression of miR-324-3p significantly promoted tumor growth *in vivo*. *P < 0.05, **P < 0.01 and ***P < 0.001 by ANOVA. (**B**) Nude mice were respectively subcutaneously transplanted with Lv-NC-Hep3B cells (*n* = 3) or Lv-anti-miR-324-3p-Hep3B cells (*n* = 3, left panel). Tumor volume (middle panel) and weight (right panel) were measured. miR-324-3p knockdown suppressed the growth of Hep3B cells *in vivo*. *P < 0.05, **P < 0.01 and ***P < 0.001 by ANOVA. (**C**) The percentage of Ki-67 positive cells in tumors arising from Lv-miR-324-3p-SMMC-7721 group was significantly higher than that in tumors arising from control group. *n* = 3, **P < 0.01 by *t* test. Scale bar: 100 μm. Original magnification was 400×. (**D**) The photomicrographs for Ki-67 staining showed that miR-324-3p knockdown inhibited Hep3B cell proliferation *in vivo*. *n* = 3, ***P < 0.001 by *t* test. Scale bar: 100 μm. Original magnification was 400×.

Additionally, we established the subcutaneous tumor models of HCC in nude mice, and the tumor growth-curve in each group was conducted. The data showed that the tumor growth was markedly promoted in SMMC-7721-miR-324-3p group compared to the control group (*P* < 0.05, Figure 4A). In contrast, miR-324-3p silencing could dramatically inhibit the growth of Hep3B cells *in vivo* (*P* < 0.05, Figure 4B). Furthermore, we measured the expression of Ki-67 in mice tumor tissues by immunohistochemistry. As expected, the expression of Ki-67 in tumor tissues with overexpressed miR-324-3p was obviously higher than those in control group (*P* < 0.01, Figure 4C). Meanwhile, the expression of Ki-67 was significantly reduced in tumor tissues with anti-miR-324-3p (*P* < 0.001, Figure 4D). Thus, the data suggested that miR-324-3p could promote tumor growth *in vivo*.

### DACT1 is a direct downstream target of miR-324-3p

**Figure 5:**
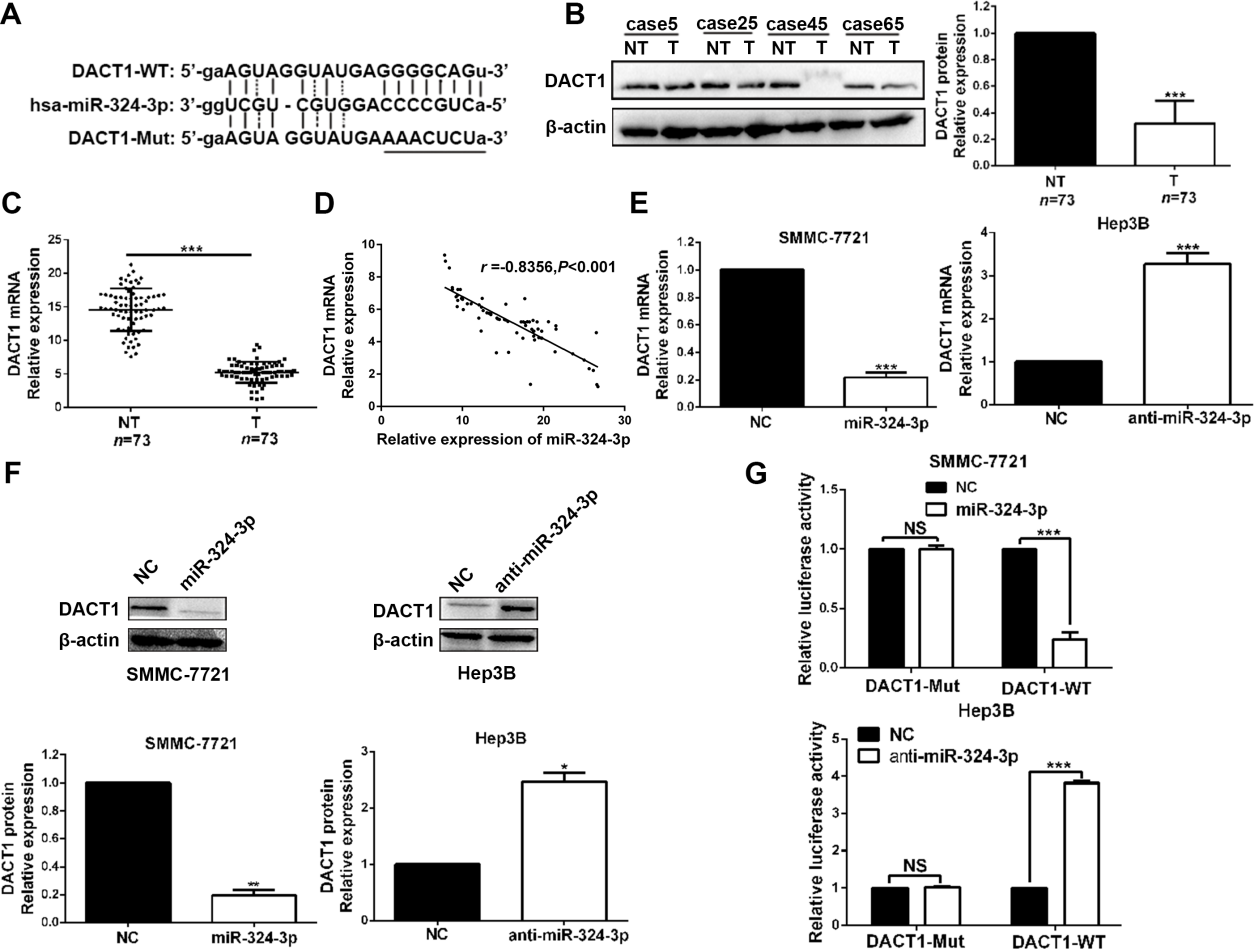
DACT1 is a direct target of miR-324-3p in HCC cells (**A**) Bioinformatics analysis revealed that 3′-UTR of DACT1 contained the highly conserved putative miR-324-3p binding sites. (**B**) and (**C**) Western blot and real-time PCR data revealed that both protein and mRNA expression of DACT1 were down-regulated in HCC tissues. *n* = 73, ***P < 0.001 by t test. (**D**) A significant inverse correlation was shown between miR-324-3p and DACT1 mRNA levels in HCC specimens. n = 73, P<0.001 by Pearson’s correlation analysis. (**E**) and (**F**) MiR-324-3p negatively regulate DACT1 mRNA and protein expression in HCC cells. *n* = three repeats with similar results, *P < 0.05, **P < 0.01 and ***P < 0.001 by *t* test. (**G**) MiR-324-3p mimics significantly suppressed the luciferase activity of DACT1-WT, while anti-miR-324-3p enhanced the luciferase activity. But, the luciferase activity of DACT1-Mut was not influenced by miR-324-3p mimics or inhibitors in HCC cells. n = three repeats with similar results, ***P < 0.001 by *t* test.

Next, we tried to search for candidate target genes of miR-324-3p through publicly available databases (TargetScan, miRanda and PicTar). Based on the analysis results (Figure 5A), we focused on DACT1, which had been described as a tumor suppressor in HCC [18],. Both Western blot and real-time PCR data revealed a significant under-expression of DACT1 in HCC tissues compared to those in adjacent non-tumor tissues (*P* < 0.001, Figure 5B and 5C). Meanwhile, we found that DACT1 expression was notably decreased in HCC cell lines compared to LO2 (*P* < 0.001, respectively, Supplementary Figure 1). In addition, a significant inverse correlation was revealed by Pearson’s correlation analysis between the expression levels of miR-324-3p and DACT1 mRNA in HCC tissues (*R* = −0.8356, *P* < 0.001, Figure 5D). Consistently, miR-324-3p overexpression could notably reduce the mRNA and protein expression of DACT1 in SMMC-7721 cells, while miR-324-3p knockdown significantly increased DACT1 level in Hep3B cells (*P* < 0.05, Figure 5E and 5F). Subsequently, dual luciferase reporter assay showed that miR-324-3p significantly suppressed the luciferase activity of reporter that carried wild type (WT) but not mutant (Mut) 3’-UTR of DACT1 (*P* < 0.001, Figure 5G). In conclusion, these data strongly suggested that DACT1 was a target of miR-324-3p in HCC.

### DACT1 mediates the effects of miR-324-3p in HCC cells

**Figure 6:**
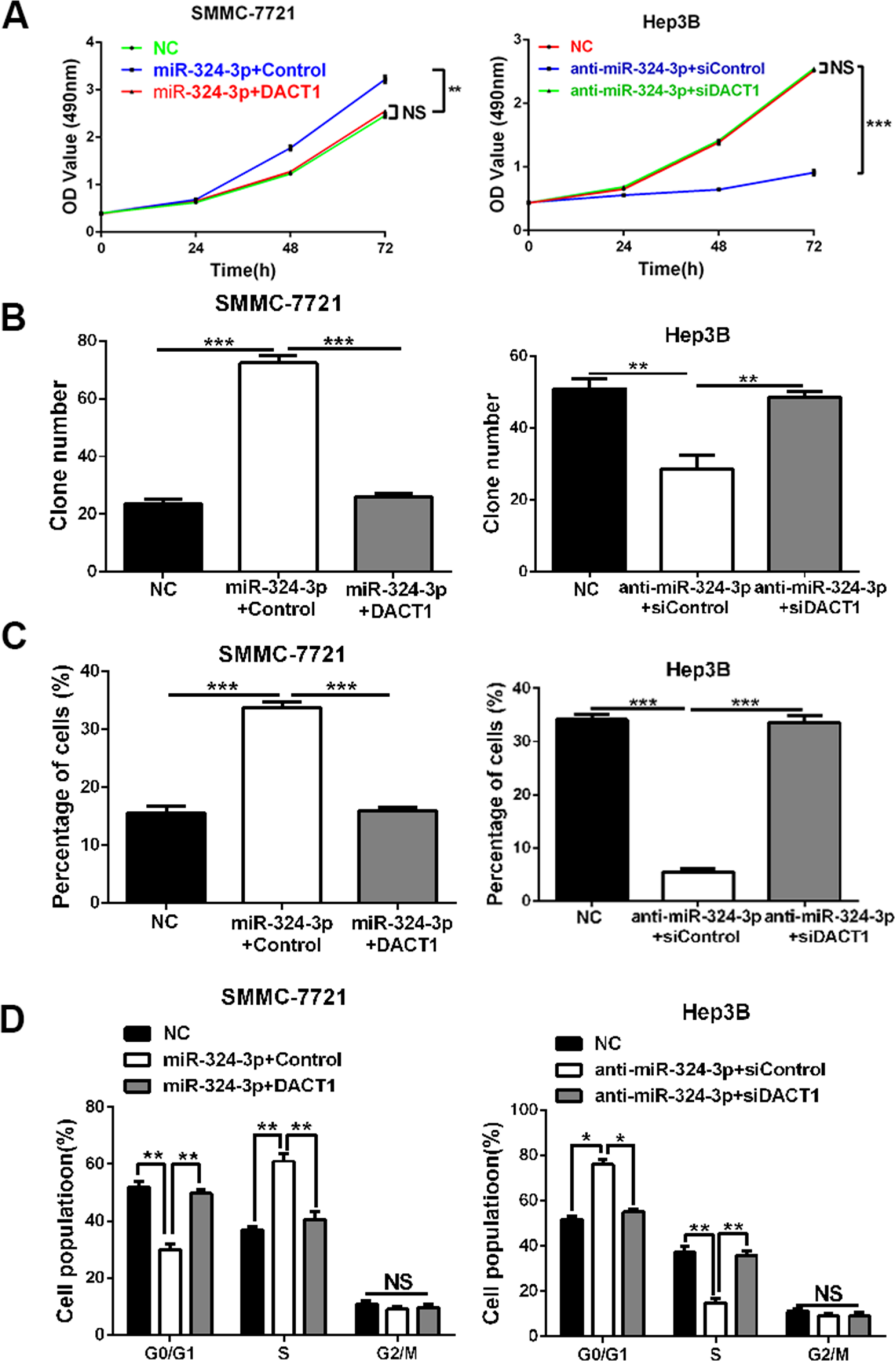
DACT1 mediates the oncogenic role of miR-324-3p in HCC cells Rescue experiments were performed using (**A**) MTT assay, (**B**) colony formation assay, (**C**) EdU cell proliferation assay and (**D**) cell cycle analysis in HCC cells. Our data revealed that DACT1 clone could reverse the effects of miR-324-3p mimics on cell viability, colony formation, proliferation and cell cycle progression of SMMC-7721 cells. In accordance, DACT1 siRNA could reverse the effects of anti-miR-324-3p in Hep3B cells. *n* = three repeats with similar results, *P < 0.05, **P < 0.01 and ***P < 0.001 by ANONA or *t* test.

Next, we attempted to determine whether DACT1 could mediate the effects of miR-324-3p in HCC cells. We conducted rescue experiments in HCC cells by co-transfection of corresponding vectors. As expected, MTT assay, colony formation assay, EdU cell proliferation assay and cell cycle analysis revealed that the promoting effects of miR-324-3p mimics on cell viability, colony formation, proliferation and cell cycle progression of SMMC-7721 cells could be largely reversed by DACT1 restoration (*P* < 0.05, Figure 6A–6D). On the contrary, DACT1 siRNA could strongly rescue the effects of miR-324-3p on cell viability, colony formation, proliferation and cell cycle progression of Hep3B cells (*P* < 0.05, Figure 6A–6D). Then, we concluded that DACT1 mediated the effects of miR-324-3p in HCC cells.

### MiR-324-3p enhances Wnt/β-catenin signaling pathway via targeting DACT1 in HCC cells

**Figure 7:**
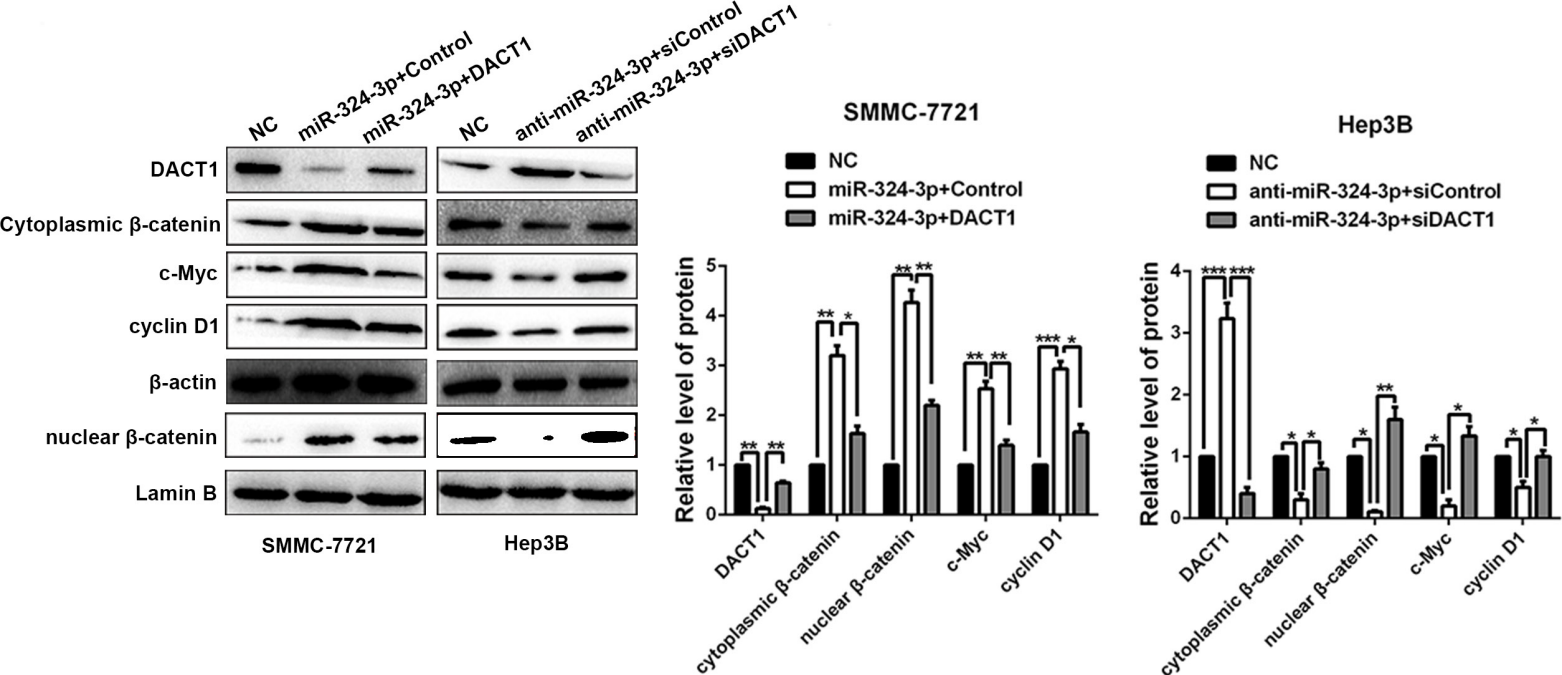
MiR-324-3p enhances activation of Wnt/β-catenin signaling pathway via targeting DACT1 in HCC cells Western blot results and according statistical graphs revealed that both cytoplasmic and nuclear β-catenin expressions were increased in SMMC-7721 cells with miR-324-3p overexpression, while decreased in Hep3B cells with miR-324-3p knockdown. Both cytoplasmic and nuclear β-catenin expressions could be reversed by DACT1 clone or siRNA compared to the control groups. Meanwhile, the expression changes of c-Myc and cyclin D1, downstream targets of Wnt/β-catenin pathway, could be positively regulated by miR-324-3p in HCC cells. These regulatory effects could be reversed by DACT1. *n* = three repeats with similar results, *P < 0.05, **P < 0.01, ***P < 0.001 by ANOVA.

Considerable researches confirmed that DACT1 could antagonize Wnt signaling pathway by binding to Dvl and promoting its degradation, which resulted in the reduced expression of both cytoplasmic and nuclear β-catenin [15, 16, 18]. Next, we explored whether miR-324-3p could increase the accumulation of both cytoplasmic and nuclear β-catenin in HCC cells. As showed in Figure 7, the results of Western blot revealed that both cytoplasmic and nuclear β-catenin expressions were increased in SMMC-7721 cells after miR-324-3p overexpression, while decreased in Hep3B cells with miR-324-3p knockdown (*P* < 0.05). Furthermore, our results showed that modulating DACT1 expression by expression plasmid or siRNA obviously reversed cytoplasmic and nuclear β-catenin expression in miR-324-3p overexpressing SMMC-7721 cells or miR-324-3p down-regulating Hep3B cells (*P* < 0.05, Figure 7). Meanwhile, c-Myc and cyclin D1, downstream targets of Wnt/β-catenin pathway, were positively regulated by miR-324-3p and negatively modulated by DACT1 in HCC cells (*P* < 0.05, Figure 7). Hence, miR-324-3p could enhanced Wnt/β-catenin signaling pathway through decreasing DACT1 expression and increasing accumulation of both cytoplasmic and nuclear β-catenin in HCC cells.

## DISCUSSION

Recently, increasing evidences have showed that miRNAs play a critical role in occurrence and progression of tumor [3, 19]. And searching for a miRNA signature might be of clinical value for diagnosis, therapy and prognosis of HCC [3, 20]. MiRNAs can exert their influences on diverse cell biological behaviors mainly through targeting their downstream genes in HCC cells [2, 21–23]. For example, miR-212 could suppress HCC growth via targeting FOXA1 [7]. And a research revealed that miR-129-2 might serve as a prognostic indicator for HCC patients and exerted a tumor suppressive role by inhibiting high mobility group box 1 (HMGB1) [24].What’s more, miR-324-3p has been suggested to be involved in tumorigenesis and tumor development by acting as an oncogene or tumor suppressor in several cancers, such as nasopharyngeal carcinoma [9, 25], colorectal cancer [26] and lung squamous cell carcinoma [10]. Especially, Wen Y *et al.* highlight that miR-324-3p is significantly overexpressed in the HBV-positive HCC patients compared with the HBV-positive cancer-free controls in both the training and validation sets [27]. However, the expression and function of miR-324-3p remain to be uncovered in HCC.

In the present study, we determined that miR-324-3p was frequently and significantly increased in HCC tissues and cell lines. In addition, elevated miR-324-3p expression was closely associated with several growth-associated malignant clinicopathologic characteristics, including large tumor size, multiple tumor nodules, advanced TNM stage. HCC patients in high miR-324-3p group had shorter 3-year OS and DFS compared to those in low miR-324-3p group. These data suggest that highly expressed miR-324-3p may be an oncogene, which plays a critical role in occurrence and progression of HCC.

To explore the functions of miR-324-3p, we conducted experiments *in vitro* and *in vivo.* Results revealed that forced expression of miR-324-3p could markedly result in enhanced cell viability, colony formation, proliferation and cell cycle progression, whereas miR-324-3p knockdown presented the contrary effects. Meanwhile, results from the subcutaneous nude mice models confirmed that miR-324-3p could promote tumor growth of HCC *in vivo*. Then, we conclud that miR-324-3p accelerates HCC cell viability, colony formation, proliferation and cell cycle progression.

It is generally known that posttranscriptionally targeting and repressing the downstream genes is the main mechanism for miRNAs to exert their functions in tumor cells [3]. Then we searched for the target genes by analyzing and summarizing the data from databases (TargetScan, miRanda and PicTar). And we focused on DACT1, which has been suggested to be a tumor suppresser in HCC by inhibiting Wnt/β-catenin signaling pathway [18]. Consistently, we found that both mRNA and protein expressions of DACT1 were notably down-regulated in HCC tissues and cell lines. Interestingly, a negative correlation between miR-324-3p and DACT1 was observed in HCC tissues. And miR-324-3p could negatively regulate the expression of DACT1 in HCC cells. In addition, dual luciferase reporter assay showed that miR-324-3p could bind to 3’-UTR of DACT1. Then, we demonstrated that miR-324-3p could directly target DACT1 in HCC cells. Furthermore, the rescue experiments revealed that DACT1 could reverse the effects of miR-324-3p on cell viability, colony formation, proliferation and cell cycle progression of HCC cells. Then, we demonstrate that DACT1 mediates the effects of miR-324-3p in HCC cells.

As described above, it has been suggested that DACT1 inhibits Wnt signaling pathway by binding to Dvl and promoting its degradation, then results in the reduced accumulation of both cytoplasmic and nuclear β-catenin [18]. Here, we confirmed that DACT1 could suppress the accumulation of both cytoplasmic and nuclear β-catenin. Next, we observed that the accumulation of both cytoplasmic and nuclear β-catenin as well as its downstream targets, c-Myc and cyclin D1, could be positively regulated by miR-324-3p in HCC cells. These regulatory effects could be reversed by DACT1. These data reveal that miR-324-3p activates Wnt/β-catenin signaling pathway via targeting DACT1 in HCC cells.

Taken together, our study report that miR-324-3p promotes tumor growth through targeting DACT1 and activation of Wnt/β-catenin pathway in HCC. This findings will improve understanding of mechanism involved in cancer progression and provide a novel target for the molecular treatment of HCC.

## MATERIALS AND METHODS

### Ethical approval

All the HCC patients signed informed consent and the experiments were approved by the Ethical Committee of First Affiliated Hospital of Xi’an Jiaotong University and complied with the guidelines and principles of the Declaration of Helsinki.

### Patients and tissue samples

Total 73 paired HCC tissues and matched non-tumor tissues were collected from patients underwent surgical resection in the First Affiliated Hospital of Xi’an Jiaotong University during the period of January 2010 to December 2013. Before resection, all of the patients had not received radiotherapy or chemotherapy. The extracted specimens were proven to be HCC tissues with pathological diagnosis. All of the fresh samples were immediately placed in liquid nitrogen container and then conserved at −80°C for further analysis.

### Real-time PCR analysis

Invitrogen TRIzol reagent (Thermofisher Scientific, Shanghai, China) was applied to isolate total RNA samples from tissues or cells. Total RNA was reverse-transcribed to cDNA with PrimeScript Reverse Transcriptase kit (Takara, Dalian, China) according to the manufacturer’s protocol. By using SYBR Green chemistry (Thermofisher Scientific), real-time PCR was implemented with the ABI 7900HT sequence detection machine (Bio-Rad, CA, USA). PCR was executed in a 10 μL reaction volume and composed of an initial denaturation step at 95°C for 30 sec, followed by amplification with 40 cycles at 95°C for 5 sec and 60°C for 30 sec, then the Melt Curve step at 65°C to 95°C and the increment at 0.5°C for 5 sec. The threshold cycle (Ct) was defined as the cycle number at which the fluorescence passed a predetermined threshold. The target and reference genes (U6 and GAPDH) were amplified in separate wells in triplicate. Primers for miR-324-3p, U6, GAPDH and DACT1 were purchased from Genecopoeia (Guangzhou, China). Gene expressions were calculated with the comparative threshold cycle (2^-ΔΔCT^) approach [28].

### Western blot

Cells were harvested and then lysed via radioimmunoprecipitation assay buffer (RIPA buffer, Thermo Fisher Scientific) for obtaining proteins. Nuclear extracts were prepared using the Nuclear Extraction Kit (Sangon Biotechology, Shanghai, China). Proteins were quantified by micro BCA method (Micro BCA Protein Assay Kit, Thermo Fisher Scientific). Subsequently, proteins were separated by SDS-PAGE and transferred onto PVDF membranes. Then, the above PVDF membranes were incubated with corresponding primary antibodies: DACT1 (1:1000, ab51260, Abcam, MA, USA), β-catenin (1:1000, ab32572, Abcam), Cyclin D1 (1:1000, ab134175, Abcam), c-Myc (1:1000, ab32072, Abcam) and β-actin (1:500, sc-130656, Santa Cruz, CA, USA). And Lamin B (1:500, sc-6216, Santa Cruz) served as a nuclear internal control. Detection was performed by enhanced chemiluminescence kit (Amersham, Little Chalfont, UK). Blots were quantified by the Bio-Rad Model GS-670 Imaging densitometer (Bio-Rad Laboratories, Inc, USA).

### Cell culture and transfection

The HCC cell lines (HepG2, Hep3B, Huh7, SMMC-7721 and Bel-7402) and the normal hepatic cell line LO2 were purchased from Chinese Academy of Sciences (Shanghai, China), then cultured with medium, which contained 10% fetal bovine serum (Gibco, USA) and 1% penicillin–streptomycin (HyClone, USA). All of the cells were maintained in a 5% CO_2_ atmosphere incubator (37°C). The miR324-3p inhibitors, miR324-3p mimics and miR324-3p negative control (NC) were obtained from GenePharma (Shanghai, China). DACT1 Human cDNA ORF Clone, DACT1 siRNAs and according Control were purchased from OriGene (OriGene Technologies, Inc, USA). According to the product specification of Lipofectamine^®^ 2000 (ThermoFisher Scientific), cells transfection was conducted when the cells confluence reached 50–70%.

### MTT assay

The viabilities of different cell groups were assessed using MTT (Sigma, St. Louis, MO, USA) assay. The cells were plated at 5 × 10^3^/well in a 96-well plate. Each group comprised ten duplicate wells. After incubation for 0, 24, 48 and 72 h with DMEM containing 10% FBS, 5 mg/mL MTT (100 µL/well) dissolved in PBS was added to each well for 4 h. After the supernatants were removed, 100 µL DMSO was added to each well. The absorbance of at least 3 individual wells of one cell type at each time point was read using a microplate reader (Bio-Rad, USA).

### Colony formation assay

Cells were cultured in 6-cm plate at a density of 200 cells/plate. After 14 days, colonies were washed with PBS, fixed with methanol for 30 min, and stained with crystal violet for 30 min. Clearly visible colonies (> 50 cells/colony) were counted as positive for growth.

### Cell cycle analysis

To measure the cell cycle, cells were collected after being transfected for 48 h, then washed with PBS, fixed with 80% ethanol overnight at −20°C. Then cells were treated with RNaseA (Sigma) for 30 min at 37°C, and followed by incubation with 20 μg/ml of propidium iodide (PI, Sigma) for 20 min at room temperature. Then FACS Calibur (BD Biosciences, Bedford, MA, USA) was applied to conduct flow cytometry analysis.

### Establishment of subcutaneous tumor model

Twelve male BALB/c nude mice (4-week-old) were divided into 4 groups with 3 mice in each group. Among them, two groups were subcutaneously injected into the flank of mice with 1 × 10^7^ Lv-miR-342-3p-SMMC-7721 cells or 1 × 10^7^ Lv-NC-SMMC-7721, the other two groups were conducted with 1 × 10^7^ Lv-anti-miR-342-3p-Hep3B cells or 1 × 10^7^ Lv-NC-Hep3B cells. And tumor volume was measured every week with a digital vernier caliper using the following formula: tumor volume = (length × width^2^)/2.28 days after implantation, mice were anaesthetized by intraperitoneal injection of 1% pentobarbital sodium before collecting samples. The protocols of animal experiments were approved by the institutional animal care and use committee of Xi’an Jiaotong University.

### Immunohistochemistry staining

The tissue sections were dewaxed and rehydrated. Then, citrate buffer was used for retrieve antigen, and hydrogen peroxide (3.0%) was used for blocking the endogenous peroxidase activity. After being blocked by 10% goat plasma, Ki-67 primary antibody (1:200, 9027S, Cell Signaling, USA) was added to the sections and incubated at 4°C overnight. Then, the biotinylated secondary antibody (ZSGB-BIO, Beijing, China) was applied for detecting the primary antibody. The sections were counterstained with hematoxylin followed by dehydrating and mounting.

### Statistical analysis

Data were presented as Mean ± SD and analyzed by GraphPad Prism 5 software (GraphPad Software, Inc, San Diego, CA, USA). Statistical methods including ANOVA, KaplanMeier method, Log-rank test, *t* test, χ^2^ test and Pearson’s correlation analysis were used respectively. *P*<0.05 was regarded to be statistically significant.

## SUPPLEMENTARY MATERIALS FIGURE


