# Supplementary material for: MiR-324-3p promotes tumor growth through targeting DACT1 and activation of Wnt/β-catenin pathway in hepatocellular carcinoma
Source: Oncotarget. 2017 Aug 7;8(39):65687–98. doi: 10.18632/oncotarget.20058 (PMC5630364; doi:10.18632/oncotarget.20058)
Supplement: Supplementary file 1 [file oncotarget-08-65687-s001.pdf]

MiR-324-3p promotes tumor growth through targeting DACT1 and activation of Wnt/β-catenin pathway in hepatocellular carcinoma

SUPPLEMENTARY MATERIALS

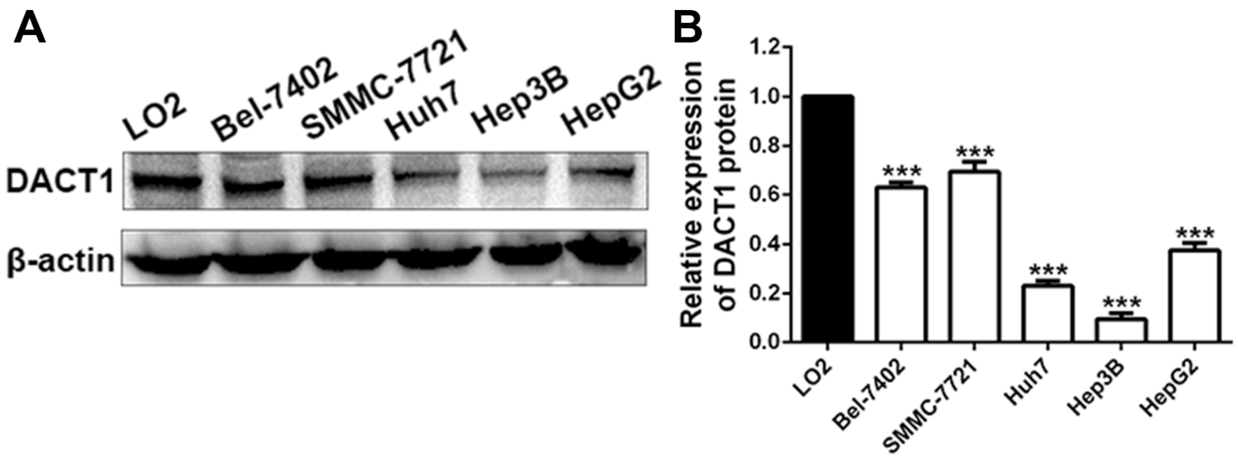

Supplementary Figure 1: DACT1 is frequently downregulated in HCC cell lines. Western blot analysis revealed that the expression of DACT1 protein in five HCC cell lines was significantly decreased compared to LO2 cells. *n* = three repeats with similar results, ***$P < 0.001$ by ANOVA.
